# ID3 deficiency alters chromatin accessibility at DSB sites and enhances vulnerability to HDAC inhibition

**DOI:** 10.1002/ijc.70400

**Published:** 2026-02-24

**Authors:** Giuditta Della Corte, Hossam Eldesouky, Julia Puchan, Sercan Öz, Elena Everatt, Ashish Goyal, Gianluca Sigismondo, Udo Oppermann, Christoph Plass, Dieter Weichenhan, Ali Bakr

**Affiliations:** ^1^ Division of Cancer Epigenomics German Cancer Research Center (DKFZ) Heidelberg Germany; ^2^ Division of Neuropathology Heidelberg University Hospital Heidelberg Germany; ^3^ Botnar Research Centre, Oxford Translational Myeloma Centre University of Oxford Oxford UK

## Abstract

The inhibitor of DNA‐binding 3 (ID3) plays a crucial role in DNA double‐strand break (DSB) repair. We previously reported that ID3 loss reduced chromatin accessibility at DNA repair gene promoters, yet its exact role in DNA repair via chromatin regulation remains elusive. Using the AID‐DIvA cell system with inducible DSBs, we show that ID3 directly regulates chromatin accessibility at DSB sites, as demonstrated by reduced chromatin accessibility and lower H3K27ac levels in ID3‐knockout (KO) cells. Loss of ID3 renders cells susceptible to histone deacetylase (HDAC) inhibition, particularly to the Class I HDAC inhibitor, leading to the accumulation of unrepaired DSBs and delayed cell cycle progression. Transcriptome and proteome analyses revealed that HDAC inhibition in ID3‐KO cells results in the downregulation of gene sets involved in the regulation of cell cycle and cell division. The synthetic lethality observed between ID3 loss and Class I HDAC inhibition underscores a novel therapeutic vulnerability in ID3‐deficient cancers, driven by compounded defects in chromatin remodeling, cell cycle, and DNA repair. Our study provides new insights into the relationship between chromatin regulation and genome stability, with implications for targeted cancer therapies.

## Graphical abstract

What's new?

Errors in DNA double‐strand break (DSB) repair can lead to mutations, chromosomal instability, and ultimately cancer. Inhibitor of DNA‐binding 3 (ID3), a transcriptional repressor, is crucial to promoting DSB repair and helping maintain genome stability. Here, the authors investigated ID3 regulation of DNA repair via chromatin accessibility in cells engineered to track real‐time repair within specific chromatin domains. Experiments show that ID3 directly regulates chromatin structure at DSB sites. Moreover, its loss compromises DNA repair, increases genomic vulnerability, and sensitizes cancer cells to Class I histone deacetylase inhibitors, revealing a potential targeted treatment strategy for tumors lacking ID3.

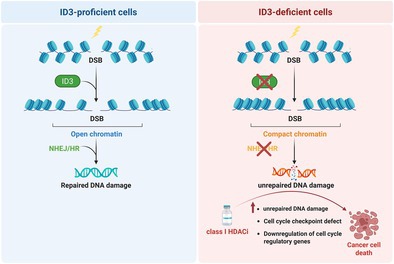

**Abbreviations**
53BP1: tumor suppressor p53‐binding protein 1
AID‐DIvA: auxin inducible degron‐DSB inducible via AsiSI
ATAC‐seq: assay for transposase‐accessible chromatin using sequencing
ATM: ataxia telangiectasia mutated
BRCA1: breast cancer 1
ChIP: chromatin immunoprecipitation
CHK1: checkpoint kinase 1
CtIP: C‐terminal binding protein 1 interacting protein
DDR: DNA damage response
DSBs: double‐strand breaks
H2AX: histone H2AX
H2B: histone H2B
H3K27ac: histone H3 lysine 27 acetylation
HDAC1: histone deacetylase 1
HDAC2: histone deacetylase 2
HDAC3: histone deacetylase 3
HDACi: histone deacetylase inhibitor
HPTMs: histone post‐translational modifications
HR: homologous recombination
ID3: inhibitor of DNA binding 3
IR: ionizing radiation
NBS1: Nijmegen breakage syndrome 1 protein
NHEJ: non‐homologous end‐joining
PARP1: poly(ADP‐ribose) polymerase 1
qPCR: real‐time polymerase chain reaction
RECQL: RecQ like helicase
RIF1: Rap1‐interacting factor 1
RNA‐seq: RNA sequencing
siRNA: small interfering RNA

## 1 INTRODUCTION

Double‐strand breaks (DSBs) are a severe form of DNA damage, where both DNA strands are broken and lack a repair template. If left unrepaired, DSBs can lead to chromosomal aberrations, mutations, and genomic instability, which contribute to cancer development. To prevent this, DSBs must be accurately repaired through the DNA damage response (DDR), which activates repair pathways and cell cycle checkpoints.^1^, ^2^ DSBs are primarily repaired via two pathways: non‐homologous end joining (NHEJ) and homologous recombination (HR). NHEJ, active throughout the cell cycle, can be mutagenic due to insertions or deletions at the repair site.^3^ In contrast, HR is an error‐free mechanism that occurs in late S or G2 phases, using homologous sister chromatids as templates for repair.^4^ The DSB repair pathway choice is controlled by a functional cell cycle dependent crosstalk between the end protection factor 53BP1/RIF1 and end resection factors BRCA1/CtIP.^5^, ^6^, ^7^, ^8^, ^9^ Chromatin modifications triggered by DSB induction enhance chromatin accessibility for these key repair proteins. However, the role of these epigenetic changes in regulating efficient and accurate DNA repair remains to be fully explored.

The inhibitor of DNA‐binding 3 (ID3) is a transcriptional repressor protein that acts by forming heterodimers with bHLH transcription factors, thus limiting their binding to DNA.^10^ Alterations of the ID3 gene such as amplifications, deletions, and mutations have been identified in several types of cancers. It has been reported that ID3 inhibits differentiation and stimulates cell proliferation via promoting cell cycle progression leading to an enhanced stem cell self‐renewal capacity.^11^ We previously reported that ID3‐deficient tumors exhibited high genomic instability and mutational signatures indicative of DNA repair defects.^12^ A study from another lab identified ID3 as one of the proteins interacting with the DNA damage response protein MDC1 in an ATM‐dependent manner following ionizing radiation (IR).^13^ Further investigations from our laboratory using comprehensive proteomic and transcriptomic analyses have revealed that ID3 plays a crucial role in DNA repair, particularly in HR. This function is mediated through two mechanisms: direct interaction with DNA repair proteins and transcriptional regulation of related genes.^14^ Our analyses revealed differential expression of DNA repair genes and altered chromatin accessibility at their promoter regions in ID3‐deficient cells. This prompted us to explore whether ID3 regulates DNA repair by modulating chromatin structure and histone post‐translational modifications (HPTMs) at regions flanking DSBs.

Here, we show that ID3 is involved in the regulation of chromatin accessibility at DSB sites, as evidenced by decreased chromatin accessibility and reduced H3K27ac enrichment in ID3‐knockout (KO) cells. Loss of ID3 renders cells highly sensitive to histone deacetylase (HDAC) inhibition, particularly to Class I HDAC inhibitors, leading to the accumulation of unrepaired DSBs and delayed cell cycle progression. Transcriptome analysis reveals that HDAC inhibition in ID3‐KO cells results in the downregulation of gene sets involved in the regulation of cell cycle and cell division. Our study provides new insights into the relationship between chromatin regulation and genome stability, with implications for targeted cancer therapies.

## 2 METHODS

### 2.1 Key resources

#### 2.1.1 Reagents

Protease inhibitor cocktail (Roche Diagnostics, Cat#11836170001). Phosphatase inhibitor cocktail (Roche Diagnostics, Cat#04906837001). Fluoromount‐G mounting medium (SouthernBiotech, Cat#0100‐01). BisBenzamide H33342 trihydrochloride (Sigma, Cat#B2261). Crystal violet (Merck, Cat# C‐0775). Puromycin (Merck, Cat# P8833). Geneticin disulfate (G418) (Roth, Cat#2039.3). Penicillin–Streptomycin (Sigma, Cat#P0781). Novex ECL HRP Chemiluminescent Kit (Invitrogen, Cat#WP20005). High sensitivity HRP Chemiluminescent Kit (Merck, Cat#WBKLS0500). PVDF membrane (Thermo Fisher, Cat#88520). Magna ChIP Protein A magnetic beads (Merck, Cat#16‐661). ChIP‐Grade Protein G magnetic beads (Cell Signaling, Cat#9006S). Agencourt AMPure XP (Beckman Coulter, Cat#A63880). PrimaQUANT™ SYBR green kit (Steinbrenner Laborsysteme, Cat#SL‐9902). MinElute PCR Purification kit (Qiagen, Cat#28006). Lipofectamine DharmaFECT 1 (Dharmacoon, Cat# T‐2001‐03). Tagment DNA TDE1 Enzyme and Buffer Kit (Illumina, Cat# 20034198). Agilent High Sensitivity D1000 ScreenTape Agilent High Sensitivity D1000 Reagents (Agilent, Cat#5067‐5585). Mocetinostat, Class I HDAC inhibitor (Selleckchem, Cat#S1122). Vorinostat (SAHA), pan HDAC inhibitor (MedChemExpress, Cat#HY‐10221). Tubastatin A, selective HDAC6 inhibitor (Selleckchem, Cat#S8049). Droxinostat, selective HDAC6/8/9/10 inhibitor (Selleckchem, Cat#S1422).

#### 2.1.2 Antibodies

Rabbit‐anti‐ID3 (Cell Signaling, Cat#9837, RRID:AB_10950969), Mouse‐anti‐beta‐Actin (Santa Cruz, Cat#sc‐47778, RRID:AB_2714189). Rabbit‐anti‐phospho‐Histone H2A.X (S139) (Abcam, Cat# ab2893, RRID:AB_303395). Rabbit‐anti‐H3K27ac (Abcam, Cat#ab177178, RRID:AB_2532991). Mouse‐anti‐IgG (Santa Cruz, Cat#sc‐2025, RRID:AB_737182). Goat‐anti‐mouse IgG‐HRP (Cell Signaling, Cat#7076P2, RRID:AB_330924). Goat‐anti‐rabbit IgG‐HRP (Cell Signaling, Cat#7074S, RRID:AB_2099233). Goat‐anti‐rabbit IgG‐AlexaFluor 488 (Molecular Probes, Cat#A11008, RRID:AB_143165), Rabbit‐anti‐HDAC1 (Abcam, Cat# ab109411, RRID:AB_10859341), Rabbit‐anti‐HDAC2 (Abcam, Cat# ab124974, RRID:AB_10973249), Rabbit‐anti‐HDAC3 (Abcam, Cat# ab137704, RRID:AB_2732844).

#### 2.1.3 Laboratory instruments

FACSCanto™ II Flow Cytometer (BD, Cat#338960). Agilent 4150 TapeStation system (Agilent, Cat#G2992AA). Amersham Imager 680 (GE Healthcare, Cat#29270769). Axioplan 2 imaging microscope (Zeiss). LightCycler® 480 (Roche, Cat#05015243001). microTUBE AFA Fiber Pre‐Slit Snap‐Cap (Covaris, Cat#80606). M220 Focused‐ultrasonicator (Covaris). Thermocycler (Eppendorf ).

#### 2.1.4 Biological resources

AID‐DIvA cells (originally U2OS cells integrated with AsiSI‐expressing vector; cells were kindly provided by Dr. G. Legube, University of Toulouse, France) were cultured as in reference 15. Upon addition of 4‐hydroxytamoxifen to the culture medium, the AsiSI enzyme is localized to the nucleus and generates several DSBs in the genome. U2OS cells, the human osteosarcoma (ATCC Cat# HTB‐96, RRID:CVCL_0042) were cultured in Dulbecco's modified Eagle's medium (DMEM) supplemented with 10% (vol/vol) fetal bovine serum (BioChrom), 100 U/mL penicillin, 100 μg/mL streptomycin (Sigma‐Aldrich). Cells were maintained in a humidified incubator with an atmosphere of 5% CO_2_ at 37°C (AID‐DIvA). Cells were routinely tested to be mycoplasma‐free. Competent *E*. *coli* DH5α were used for transformations. All human cell lines have been authenticated using STR (or SNP) profiling within the last 3 years.

### 2.2 siRNA and plasmid transfection

A set of four siGENOME upgrade siRNAs were obtained from Dharmacoon (Table S1, Supporting Information), pooled together and transfected using Lipofectamine DharmaFECT1 (Dharmacon) according to the manufacturer's protocol. Plasmid transfections were carried out using TransITLT1 (Mirus Bio) according to the manufacturer's protocol. Plasmids were transfected 48 h after siRNA treatment.

### 2.3 DNA‐damage induction in AID‐DIvA cells

Wild‐type (WT) or ID3‐KO AID‐DIvA cells were treated with 300 nM 4‐hydroxytamoxifen (4OHT) for 6 h to induce AsiSI introgression into the nucleus and generation of DSBs. DSB induction was terminated by Auxin supply as described previously.^15^ The type of repair pathway, HR or NHEJ, acting at the different DSB sites has been described previously.^15^

### 2.4 Whole‐cell protein extracts and Western blotting

Whole‐cell protein lysates were extracted using RIPA buffer as previously described.^5^ Protein concentrations were determined using a BCA assay (Sigma‐Aldrich) as previously described.^14^ Protein extracts were prepared in Laemmli buffer and denatured at 95°C then loaded on SDS gel for western blotting. Separated proteins were transferred to a PVDF membrane, blocked at RT for 1 h in 5% skimmed milk in TBS‐Tween and incubated overnight at 4°C with primary antibodies. Membranes were then washed three times and incubated with HRP‐conjugated secondary antibodies at RT for 1 h. Detection was done by the HRP Chemiluminescent Substrate Reagent Kit Novex ECL (Invitrogen) or high sensitivity Kit (Merck Millipore). Measurement is performed using the Amersham imager 680 GE (Healthcare).

### 2.5 Epigenetic compound screening and cell viability assay

We screened a library of 172 epigenetic compounds^16^ in ID3‐KO and wild‐type (WT) cells. Cells were cultivated in 96‐well plates and treated with drug solutions or a vehicle for 48 h and left another 24 h for recovery following medium exchange, then relative viability was determined using Cell Titer Blue assay as described previously.^17^, ^18^ CellTiter Blue reagent (#G8081, Promega, Madison, WI) was added to determine the metabolic capacity of the cells, incubated for 2 h, and the fluorescence intensity was measured at a 560 nm excitation wavelength and a 590 nm emission wavelength using a SpectraMax M5 plate reader.

### 2.6 Colony formation assay

2000 cells were seeded into 6‐well plates and incubated overnight at 37°C. Cells were then treated with Class I HDAC inhibitor (Mocetinostat). The medium was exchanged after 24 h and plates were incubated at 37°C, 5% CO_2_, and left for 14 days. Afterward, the medium was removed, and the colonies were fixed using 70% ethanol and stained using 1% crystal violet. The colonies were finally counted. The data are presented as the mean ± SEM value in three independent experiments.

### 2.7 Immunofluorescence analysis

Assays were performed as previously described.^14^ 2 × 10^5^ cells were seeded onto comet slides (R&D systems), incubated overnight then treated with 1 μM Class I HDAC inhibitor (Mocetinostat). Cells were fixed after the indicated time points in 4% paraformaldehyde, permeabilized in 0.15% PBS–Triton X100, and blocked (1% BSA and 0.15% glycine in PBS). Cells were incubated overnight at 4°C with primary antibody. Slides were washed in PBS, permeabilized, and blocked. Next, cells were incubated for 1 h at RT in the dark and subsequently washed in PBS and DNA was stained using bis‐Benzimide (Sigma‐Aldrich) in Tris–HCl for 3 min. The mounting medium was added before sealing with a cover slide. Fluorescent images were taken by using the Zeiss Axioplan 2 imaging microscope and foci were automatically counted by the Metafer4 system with a magnification of 400×.

### 2.8 Cell cycle analysis

Assays were performed as previously described.^19^ Briefly, cells (approximately 400,000) were treated by Class I HDAC inhibitor (Mocetinostat) and collected and fixed with 75% ethanol at the indicated time points. Cells were then washed with PBS and permeabilized in 0.1% PBS–Triton X100. RNA was digested by RNase Athen cells were stained by 50 μg/mL propidium iodide (PI). The stained cell suspension is filtered and analyzed using FACSCanto™ II Flow Cytometer. The flow cytometry raw data were analyzed using FACS Diva software. The gating strategy was applied as follows: first, cells were gated based on SSC‐A vs. FSC‐A to define population P1. This population was subsequently gated on 616/23 PI BL‐B‐H vs. 616/23 PI BL‐B‐A to define population P2. Finally, P2 was represented in a histogram of count vs. 616/23 PI BL‐B‐A. The percentage of cells in each cell cycle phase is calculated based on the gating and the histogram data.

### 2.9 Chromatin immunoprecipitation and qPCR

DSB induction and termination were done as described above. Chromatin immunoprecipitation and qPCR (ChIP‐qPCR) was performed as described previously.^20^ Cells were cross‐linked with formaldehyde (1%) and cell pellets were obtained and washed in ice‐cold PBS. Nuclei were isolated and then transferred into Adaptive Focused Acoustics (AFA) for chromatin shearing. 25 μg of the sheared chromatin was incubated with 2–5 μg of antibody overnight at 4°C. Pre‐blocked magnetic beads were added to the antibody‐chromatin mixture and incubated for 3 h at 4°C on a rotator. Beads were washed and incubated with an Elution buffer supplemented with proteinase K, then treated with RNAse and stored at 4°C. DNA was then eluted and purified using Ampure beads (Agencourt AMPure XP) at room temperature (with a ratio 1:1.4). Then washed twice with 80% ethanol, dried, and finally eluted in dH2O at room temperature. For qPCR, proximal (80 bps) or distal (800 bps) primers for the indicated DSBs were used (Table S1). The qPCR was performed using primaQUANT™ SYBR green kit.

### 2.10 Assay for transposase‐accessible chromatin using sequencing (ATAC‐seq)

Wild‐type and ID3‐KO AID‐DIvA cells were treated with 300 nM 4‐hydroxytamoxifen (4‐OHT) for 6 h at 37°C to induce AsiSI localization to the nucleus and generate DSBs (3 biological replicates for each condition). Next, 50,000 viable cells were collected before and after 4‐OHT treatment and lysed in cold lysis buffer (10 mM Tris–HCl pH 7.4, 10 mM NaCl, 3 mM MgCl_2_) containing 0.1% digitonin, 1% NP40, 0.1% Tween‐20, and incubated on ice for 5 min to isolate the nuclei. Subsequently, nuclei were pelleted by centrifugation at 1000*g* for 5 min at 4°C. The nuclei pellets were resuspended in transposition buffer (Illumina) containing 0.1% digitonin, 0.1% Tween‐20, and 2.5 μL of Tagment DNA Enzyme 1 (Illumina) was added onto samples. The samples were mixed in a thermomixer at 1000 rpm for 30 min at 37°C. 5M guanidium thiocyanate were added to the samples to stop the tagmentation reaction. DNA was then purified by AMPure XP PCR Purification (Beckman Coulter, A63880). The libraries were amplified by real‐time quantitative PCR adding of NEBNext® High Fidelity 2X PCR mix, 10 μM Custom Nextera PCR Forward Primer, 10 μM Custom Nextera PCR Barcode Primers, and SYBR Green (1X final concentration). The library amplification program was as follows: 5 min at 72°C for gap repair, 30 s at 98°C for initial melting, 10 s at 98°C, 30 s at 63°C, and 30 s at 72°C for amplification. Libraries were subsequently purified using 1.4X AMPure beads. DNA concentration was measured using the Qubit instrument, and Quality control was performed on a Tape station using the Agilent High Sensitivity DNA Kit. Sequencing was performed at the DKFZ Genomics Core Facility using the Nextseq 550 Paired‐End 75 bp.

#### 2.10.1 ATAC‐seq data analysis

Sequencing reads were processed using the CWL‐based ATAC‐seq workflow.^21^ Then, IDR analysis (v 2.0.3) was conducted to identify reproducible peaks across biological replicates, applying an IDR threshold of ≤0.05. Differential accessibility analysis was carried out using Diffbind (v 3.4.11), enabling the detection of regions with statistically significant differential accessibility between conditions or groups. The sequencing coverage and quality statistics for each sample are summarized in Table S5.

### 2.11 RNA isolation and gene expression analysis by RNA sequencing

Cells (WT and ID3‐KO) were treated with Class I HDAC inhibitor (Mocetinostat) for 24 h (3 biological replicates for each condition), then samples were either collected (before recovery) or medium was exchanged with fresh DMEM medium without Mocetinostat and incubated for another 24 h (after recovery). Cells were collected and RNA extraction was performed as described previously.^14^, ^22^ Sequencing libraries were prepared by the Genomics and Proteomics Core Facility (DKFZ, Heidelberg) from total RNA using the Illumina TrueSeq Stranded Total RNA Library Prep Kit according to the manufacturer's instructions. Samples were sequenced in a paired‐end setting (100 bp) on an Illumina NovaSeq 6000 machine for sequencing.

#### 2.11.1 RNAseq data analysis

Data was processed by the Omics IT and Data Management Core Facility (DKFZ, Heidelberg), using the Roddy RNA‐seq Workflow. Default parameters were used unless mentioned otherwise. Sequences were aligned to the human reference genome (hg19/GRCh37) by applying the software STAR.^23^ Gene counts were generated with featureCounts^24^ and the gene annotation v.29 lift 37. For the identification of differentially expressed genes, the R library DESeq2 was used.^25^ Genes with an adjusted *p*‐value <.05 were defined as significantly differentially expressed genes (DEG). The sequencing coverage and quality statistics for each sample are summarized in Table S6.

### 2.12 Statistical analyses

Unless stated, GraphPad Prism v5 software was used to create graphs, perform statistical tests, and calculate *p*‐values. Statistical analyses for ATAC‐seq were performed using R version 4.3.^26^

### 2.13 Mass spectrometry sample preparation and data acquisition

Nuclear protein lysates from ID3‐KO and WT cells post‐mocetinostat treatment were extracted and analyzed for mass spectrometry as previously described.^22^

#### 2.13.1 Mass spectrometry data processing, analysis and visualization

RAW data were processed with Maxquant software (v 1.5.1.2) including the Andromeda search engine.^27^, ^28^ Peptide identification was performed as previously described.^14^ FDR was set to 1% at both protein and peptide levels. Match between runs option was enabled, Label‐Free Quantification (LFQ) and iBAQ calculated. For further protein analysis, Perseus free software was used.^29^ Two‐sided *t*‐test statistics were used for the generation of the volcano plots based on LFQ log10 values of expressed proteins. FDR was 0.05 and S0 constant was 0.1. Pathway enrichment analysis was done using the Metascape resource.^30^

## 3 RESULTS

### 3.1 ID3 regulates chromatin accessibility and histone modifications at DSBs

**FIGURE 1:**
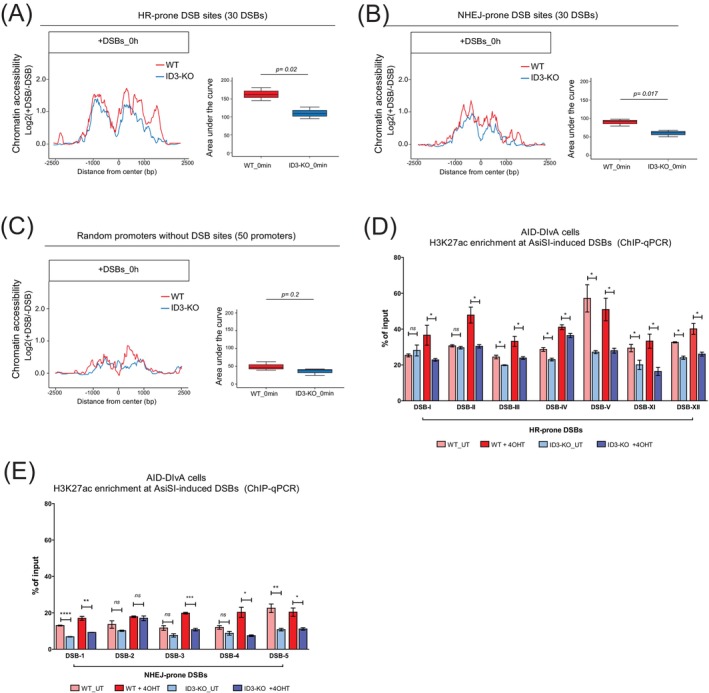
ID3 regulates chromatin accessibility and histone modifications at DSBs. (A) Profile plot showing the differentially chromatin accessibility in WT and ID3‐KO cells at HR‐prone and after DSBs induction (left panel). Box plots showing the area under the curve reflecting the quantification of differential accessibility, at HR‐prone DSBs, *n* = 3 data are presented as mean ± SD, Student's *t* test (right panel). (B) Profile plot showing the differentially chromatin accessibility in WT and ID3‐KO cells at NHEJ‐prone and after DSBs induction (left panel). Box plots showing the area under the curve reflecting the quantification of differential accessibility, at NHEJ‐prone DSBs, *n* = 3 data are presented as mean ± SD, Student's *t* test (right panel). (C) Profile plot showing the differentially chromatin accessibility in WT and ID3‐KO cells at control promoter regions lacking DSBs in their vicinity and after DSBs induction (left panel). Box plots showing the area under the curve reflecting the quantification of differential accessibility, at control promoters, *n* = 3 data are presented as mean ± SD, Student's *t* test (right panel). (D, E) Enrichment of H3K27ac at the HR‐prone and NHEJ‐prone DSBs, respectively, in WT and ID3‐KO cells, measured by ChIP‐qPCR. *n* = 3 independent experiments; data are presented as mean ± SD, Student's *t* test. Statistical significance is presented as: *p < .05, **p < .01, ***p < .001, ****p < .0001, ns = not significant.

Building on our previous findings demonstrating ID3‐dependent chromatin remodeling at gene promoters,^14^ we hypothesized that ID3 orchestrates chromatin dynamics at DNA damage sites to facilitate repair. To examine this, we performed ATAC‐seq to evaluate DNA damage‐induced changes in chromatin accessibility in wild‐type (WT) and ID3‐knockout (KO) cells following DSB induction. Knockout efficiency was validated by Western blot analyses (Figure S1A). Analysis included both DSB sites and control promoter regions lacking DSBs in their vicinity. WT cells exhibited a general increase in chromatin accessibility surrounding both HR‐ and NHEJ‐prone DSBs upon DNA damage. In contrast, ID3‐deficient cells showed a marked reduction in DNA damage‐induced chromatin accessibility relative to WT cells at both HR‐ and NHEJ‐prone DSB sites (Figure 1A,B). Notably, at control promoters without nearby DSBs, WT and KO cells displayed a very marginal increase in chromatin accessibility following DNA damage, and no significant difference between the two genotypes was observed (Figure 1C). These results indicate that while a marginal global increase in chromatin accessibility may occur upon DNA damage, this effect is not genotype‐dependent and remains substantially smaller than the robust response observed at DSB sites, where ID3 loss showed a significant reduction in chromatin accessibility compared to WT. Based on these data, we conclude that ID3 specifically facilitates the establishment of an open chromatin configuration at DSBs in response to DNA damage.

To further explore the underlying mechanism, we profiled histone H3 lysine 27 acetylation (H3K27ac), a hallmark of open chromatin, around DSBs. Upon DNA damage induction, WT cells showed enhanced H3K27ac enrichment near both HR‐ and NHEJ‐prone DSBs, whereas this response was abolished in ID3‐deficient cells (Figure 1D,E), supporting our ATAC‐seq findings. Notably, ID3 depletion also reduced basal H3K27ac levels at specific genomic loci prior to DSB induction, suggesting that ID3 contributes to establishing a permissive chromatin state at certain sites in a locus‐dependent manner that may depend on the genomic context.

### 3.2 Epigenetic vulnerability screen identifies class I HDAC dependency

The observed reduction in chromatin accessibility and H3K27ac levels at DSB sites in ID3‐KO cells (Figure 1A–D) prompted us to investigate whether compromised chromatin regulation in these cells could be exploited therapeutically. Given ID3's established role in promoting DNA repair, we hypothesized that ID3‐deficient cells would exhibit higher dependence on compensatory epigenetic regulators to maintain chromatin homeostasis. Therefore, epigenetic inhibitors could increase genomic instability in ID3‐deficient cells, leading to selective elimination of them, offering a targeted therapeutic strategy for tumors with low ID3 expression.

**FIGURE 2:**
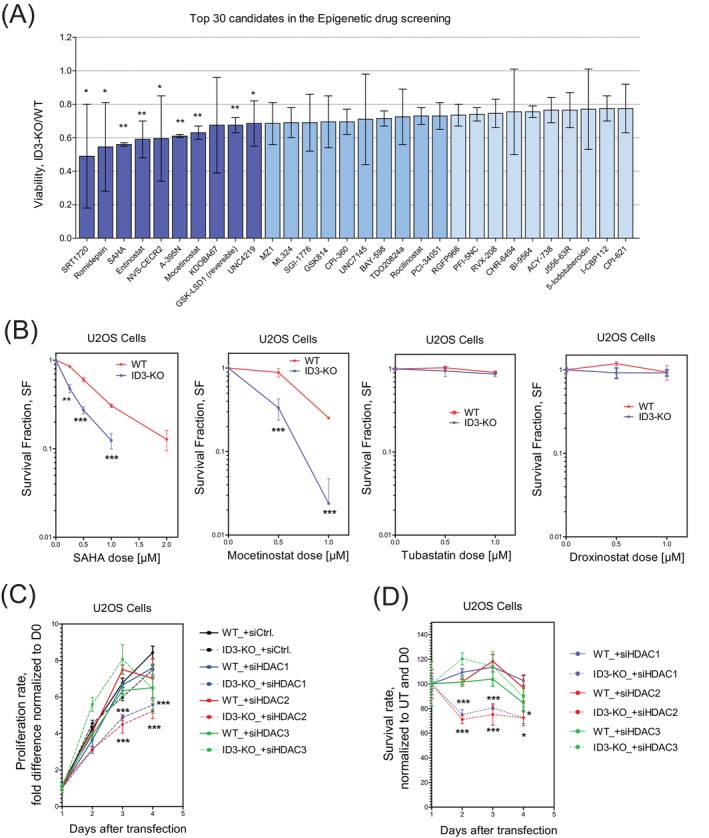
Epigenetic vulnerability screen identifies Class I HDAC dependency. (A) Bar plot showing the top 30 candidates in the Epigenetic drug screening. (B) Clonogenic survival assay of U2OS cells transfected either wild‐type (WT) or ID3‐knockout (ID3‐KO) treated with the indicated dose of HDAC inhibitors. (C, D) Cell viability assay showing the proliferation rate and survival analysis, respectively, of U2OS cells after transfection with the indicated siRNAs. *n* = 3 independent experiments; data are presented as mean ± SD. Student's *t* test was performed. Statistical significance is presented as: *p < .05, **p < .01, ***p < .001, ****p < .0001, ns = not significant.

To test this, we screened a library of 172 epigenetic compounds^16^ in ID3‐KO and wild‐type (WT) U2OS cells. Knockout efficiency was validated by Western blot analyses (Figure S1B). ID3‐KO cells showed pronounced sensitivity to several histone deacetylase inhibitors (HDACi), an inhibitor of polycomb repressive complex 2 (PRC2), and lysine demethylase inhibitors (Figure 2A and Table S2). Among the top ten identified compounds, five were classified as HDAC inhibitors, including mocetinostat that is categorized as class I HDAC inhibitor. This indicates that ID3 depletion may enhance the efficacy of these identified epigenetic drugs, highlighting potential therapeutic strategies targeting ID3‐deficient tumors. To validate these results, we analyzed the survival and the clonogenic ability of ID3‐KO cells after treatment with either pan‐HDACi (SAHA), class I HDACi (mocetinostat), or other HDACi that target different classes (tubastatin and droxinostat). SAHA and mocetinostat induced synthetic lethality in KO cells, while there was no effect on survival by tubastatin or droxinostat (Figure 2B). Further analysis of the proliferation rate demonstrated a significant reduction in the proliferation and survival of ID3‐KO cells when treated with mocetinostat (Figure S1C,D). Reintroduction of ID3 into ID3‐KO cells restored resistance to mocetinostat (Figure S1C,D). Moreover, depletion of either HDAC1 or HDAC2, but not HDAC3 triggers impaired proliferation and survival of ID3‐KO cells (Figure 2C,D). The Knockdown efficiency of HDAC1, HDAC2, and HDAC3 was validated by Western blot analyses (Figure S1E–G). This suggests that targeting HDAC1 or HDAC2 can be effective to eliminate ID3‐deficient tumors.

### 3.3 Class I HDAC inhibition exacerbates DNA damage in ID3‐deficient cells

**FIGURE 3:**
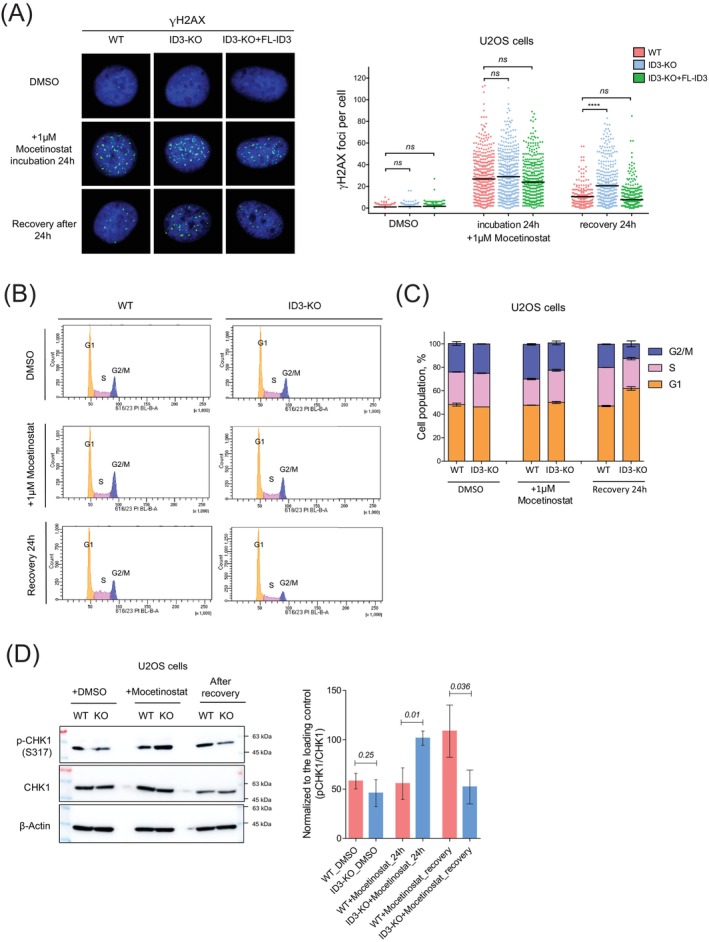
Class I HDAC inhibition exacerbates DNA damage in ID3‐deficient cells. (A) Representative micrographs and quantification of γH2AX foci in U2OS cells following treatment with class I HDACi, ca. 500 cells were counted at indicated time points. Data are presented as mean ± SEM; Student's *t* test was performed. (B, C) Histograms and bar plot showing the distribution of cell populations through the cell cycle phases. *n* = 3, data are presented as mean ± SD; Student's *t* test. (D) Western blot expression analysis of p‐CHK1 in WT and ID3‐KO cells treated with class I HDACi. Box plots showing the quantification of three independent experiments (right panel), data are presented as mean ± SD; Student's *t* test.

Higher sensitivity and reduced proliferation of ID3‐depleted cells after treatment with class I HDACi (Figure 2B,C) reflect a critical vulnerability rooted in accumulation of unresolved DNA damage. The analysis of γH2AX foci revealed higher accumulation of DNA damage in ID3‐depleted cells after recovery from mocetinostat treatment compared to WT (Figure 3A), with damage persistence rescued by ID3 reconstitution, confirming ID3's role in mitigating genomic instability.

The accumulation of unrepaired DSBs in ID3‐KO cells following Class I HDAC inhibition triggered aberrant cell cycle progression, reflecting dual defects in damage signaling and checkpoint control. Mocetinostat‐treated ID3‐KO cells exhibited reduced G2/M phase cells that can be caused by several factors, and the cells accumulated in the G1 phase after 24 h of recovery compared to WT cells (Figure 3B,C), consistent with checkpoint adaptation, a process typically induced by persistent DSBs that allow cells to bypass cell cycle arrest. This was accompanied by reduced phosphorylation of checkpoint kinase 1 (CHK1) at 24 h of recovery (Figure 3D). The diminished CHK1 activation likely accounts for the reduced G2/M population, as CHK1 is required for checkpoint signaling at this stage. The concomitant accumulation of cells in G1 may be partially explained by progression from G2/M into G1, where they subsequently arrest. However, the potential involvement of CHK2, the kinase primarily mediating the G1 checkpoint in response to DSBs, remains unresolved, as we were unable to demonstrate CHK2 phosphorylation by western blot, most likely due to insufficient antibody performance.

### 3.4 HDAC inhibition disrupts ID3‐dependent transcription of cell cycle genes

To delineate the molecular basis of HDACi‐induced synthetic lethality in ID3‐deficient cells, we analyzed transcriptional changes following mocetinostat treatment. The primary focus was to investigate the differential response and recovery mechanisms of ID3‐KO versus WT cells following mocetinostat treatment. To rigorously capture the treatment effects while accounting for baseline differences inherent to each genotype, we compared KO + HDACi samples with untreated KO controls, and analogously WT + HDACi samples to their untreated counterparts. This approach allowed us to account for the steady state of each genotype and avoided confounding effects arising from inherent transcriptional differences between KO and WT cells prior to treatment.

**FIGURE 4:**
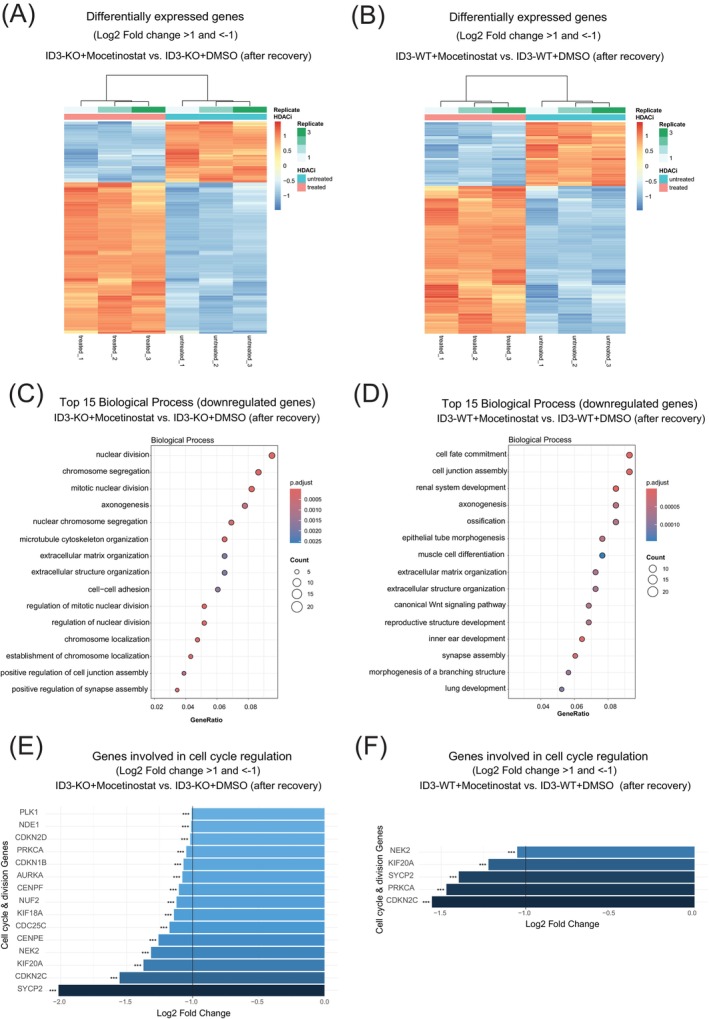
HDAC inhibition disrupts ID3‐dependent transcription of cell cycle genes. (A, B) Heat maps showing HDACi‐induced changes in gene expression in KO and WT, respectively. (C, D) Gene set enrichment analysis of the significantly downregulated genes in KO and WT, respectively, after treatment with class I HDACi. (E, F) Bar plot showing the downregulation of the genes involved in the regulation of cell cycle and division in KO and WT, respectively. *n* = 3 independent experiments; data are presented as mean ± SD, Student's *t* test. Statistical significance is presented as: *p < .05, **p < .01, ***p < .001, ****p < .0001, ns = not significant.

RNA‐seq revealed distinct dysregulation in ID3‐KO cells post‐recovery, with 838 genes upregulated (adj. *p* < .05, Log2FC >1) and 338 downregulated (adj. *p* < .05, Log2FC < −1), whereas a similar comparison in WT cells revealed that 812 genes were upregulated and 352 genes were downregulated (Figure 4A,B and Tables S3 and S4). Upregulated genes in both genotypes showed significant enrichment for leukocyte activation, leukocyte migration, and leukocyte proliferation‐related pathways. This common response suggests that HDAC inhibition triggers a conserved immune‐associated transcriptional program independent of ID3 status (Figure S2A,B). Pathway analysis of downregulated genes in KO cells identified profound suppression of mitotic cell division, chromosome segregation, and cell cycle regulation, while this transcriptional change in the mentioned pathways was absent in WT cells (Figure 4C,D). This aligns with prior observations of G1 accumulation (Figure 3C) and checkpoint exhaustion (pCHK1 decline), although we acknowledge that these associations are correlative and do not establish causality between proliferation arrest and loss of checkpoint regulators. Further analyses of cell cycle and division regulation genes revealed a downregulation of 15 key regulators of cell cycle and division, while only 5 genes were downregulated in WT cells (Figure 4E,F). For samples collected prior to recovery, pathway analysis of both downregulated and upregulated genes in KO cells revealed enrichment patterns that closely resembled those observed in WT cells (Figure S2C–F). This finding indicates that under acute treatment exposure, both genotypes exhibit similar transcriptional responses, while distinct gene expression differences emerge only after the recovery phase. Taken together, these results indicate that the absence of ID3 leads to the loss of a transcription regulatory axis responsible for mitotic cell division and cell cycle regulation genes under treatment with class I HDACi.

To further confirm the molecular basis of HDACi‐induced synthetic lethality at the protein level, we performed global proteomic profiling of ID3‐KO and WT cells post‐mocetinostat treatment. ID3‐KO cells exhibited pronounced dysregulation, with 174 proteins upregulated and 51 downregulated (adj. *p* < .05, Log2FC >1), compared to modest changes in WT cells with 70 proteins upregulated and 39 proteins downregulated (Figure S3A,B). Downregulated proteins in KO cells were enriched for mitotic cell division and DNA metabolic processes, which were not enriched in WT cells (Figure S3C,D). This suppression mirrors transcriptomic deficits in cell cycle genes (Figure 4E) and aligns with prior observations of G1 arrest (Figure 3C) and checkpoint collapse (pCHK1 decline), confirming ID3's role in sustaining proliferative and repair machinery under epigenetic stress. Upregulated proteins in both genotypes showed significant enrichment for several immune‐related pathways (Figure S3E,F). This indicates that HDACi broadly activates immune pathways through histone hyperacetylation, independent of ID3 status.

## 4 DISCUSSION

Several studies, including our own, demonstrated the involvement of ID3 in the regulation of the DNA damage response.^12^, ^13^, ^14^ Loss of ID3 leads to genomic instability and reduced chromatin binding of key DSB repair factors such as NBS1, RAD50, and RECQL following induction of DNA damage.^14^ Our previous analyses revealed DNA damage‐induced interactions of ID3 with several chromatin modifiers (belonging to GO:0071103 DNA conformation change) and altered chromatin accessibility at the promoter regions of DNA repair genes in ID3‐deficient cells.^14^ Moreover, another study reported a repressive effect of ID3 loss on chromatin accessibility in ID3‐null mice, which can be attributed to a possible interaction of ID3 with various specific transcription factors (TFs).^31^ This prompted us to explore whether ID3 regulates chromatin structure and HPTMs at regions flanking DSBs and subsequently promotes DNA repair. Our current study establishes ID3 as a critical regulator of chromatin dynamics at DSB sites, where it facilitates DNA repair by maintaining chromatin accessibility and H3K27ac enrichment, which can subsequently promote the recruitment of DNA repair factors. This is consistent with our previous study that reported ID3 promotes DSB repair by regulating the recruitment of the MRN complex and RECQL helicase under ATM‐dependent phosphorylation of ID3.^14^ These data expand the functions of ATM kinase activity to regulate chromatin accessibility at the DSB via ID3 and reinforce published reports that ATM's role is not restricted only to the recruitment of early‐stage DNA repair factors,^32^ and it is equally important to the chromatin organization during DNA repair.^33^, ^34^, ^35^, ^36^ The observed reduction in chromatin accessibility and H3K27ac levels at DSB sites in ID3‐KO cells suggests a failure to establish a repair‐permissive chromatin state and subsequent reduction in DNA repair efficiency. This defect correlated with delayed recruitment of several key DSB repair factors as well as the accumulation of ID3 itself at the DSB sites observed in previous studies including ours.^13^, ^14^ Accumulation of H3K27ac at DNA damage sites is regulated by several factors, most notably the histone acetyltransferases p300 and CBP. Studies showed that both enzymes are required for histone acetylation at DNA damage sites and for the recruitment and activation of DNA repair proteins.^37^, ^38^ Loss of p300/CBP, or their failure to efficiently locate to DSBs, can lead to decreased H3K27ac and impaired DNA repair. Our current data do not demonstrate transcriptional downregulation of p300/CBP, suggesting that the reduction in H3K27ac is more likely due to failed recruitment or altered activity at DSBs in the absence of ID3, rather than reduced expression. Collectively, we conclude that ID3 is a critical regulator of chromatin dynamics at DSB sites, potentially through modulation of histone acetylation patterns, thereby facilitating efficient DNA repair processes. This can be mediated by DNA damage‐induced interactions of ID3 with several chromatin modifiers identified in our previous study.

These results highlight a critical vulnerability that could be leveraged therapeutically, since ID3 loss is a driver of DNA repair deficiency and genomic instability. This genomic instability creates a dependency on compensatory epigenetic regulators to maintain chromatin homeostasis, a vulnerability that can be targeted with precision. Our findings that ID3 deficiency confers synthetic lethality with Class I HDAC inhibition reveal a novel and actionable therapeutic vulnerability in ID3‐deficient cancers. The specificity of this effect to ID3‐deficient cells is underscored by the observation that reconstitution of ID3 rescues sensitivity to HDAC inhibitor (HDACi), highlighting a unique dependency in tumors with low ID3 expression. These results extend previous work demonstrating the role of ID3 in maintaining genome stability.

This vulnerability can be explained by several mechanisms, for example, it may arise from compounded defects in chromatin remodeling and DNA repair. ID3‐KO cells exhibit compromised chromatin accessibility and reduced H3K27 acetylation, defects that are exacerbated by additional chromatin remodeling disruptions following HDAC1/2 inhibition. While HDACi typically increase global histone acetylation and promote chromatin decompaction, their effects at DSB sites can be context‐dependent. Notably, published reports showed that HDACi inhibit several acetyltransferases involved in DNA repair, which are critical for maintaining local histone acetylation at DSBs to establish an accessible chromatin state required for efficient repair.^39^ Consequently, HDAC1/2 inhibition in ID3‐KO cells can alter chromatin organization, potentially causing aberrant local chromatin compaction or impaired accessibility near break sites, thereby collapsing DSB repair and leading to catastrophic accumulation of unrepaired breaks. Further investigation of chromatin accessibility at DSB sites in ID3‐KO after HDAC inhibition is warranted.

Another possible explanation for our findings is the involvement of HDAC inhibition in the inactivation of NHEJ, which triggers synthetic lethality in the context of the established HR deficiency in ID3‐KO cells. Published studies confirm that ID3‐deficient cells display pronounced defects in HR with additional impairment of NHEJ, though NHEJ activity is not completely abolished.^13^, ^14^ As a result, ID3‐deficient cells are highly sensitive to agents that induce DSBs due to persistent DNA lesions and genomic instability in the absence of efficient repair pathways. Importantly, inhibition or depletion of HDAC1/2 has been associated with impaired NHEJ efficiency, leading to hypersensitivity to DNA‐damaging agents and prolonged DNA‐damage signaling.^40^ Thus, treatment of ID3‐deficient cells with class I HDAC inhibitors further exacerbates their DNA repair deficiency by targeting the residual NHEJ capacity. Mechanistically, synthetic lethality arises in these cells because HR is already compromised due to ID3 loss, and HDAC inhibition effectively abolishes the backup NHEJ pathway, ultimately triggering cell death.

A further possible explanation is supported by our results showing that the accumulation of unrepaired DSBs in ID3‐KO cells following exposure to the class I HDAC inhibitor mocetinostat triggered aberrant cell cycle progression. We observed reduced G2/M populations and G1 arrest after 24 h of recovery from mocetinostat, reflecting dual defects in DNA damage signaling and checkpoint control. As these cells cannot tolerate the persistence of unrepaired DNA lesions, selective cell death in ID3‐deficient populations ensues. The cellular response to DNA damage primarily depends on two distinct kinase signaling pathways: the ATM–CHK2 and ATR–CHK1 cascades. These pathways coordinate mechanistically distinct checkpoint responses that facilitate DNA repair and promote cell survival. The ATM–CHK2 axis predominantly mediates the G1 checkpoint by triggering G1 arrest in response to DSBs, whereas the ATR–CHK1 pathway primarily responds to replication‐associated single‐stranded DNA regions formed during S and G2 phases, thereby mediating G2/M checkpoint activation.^41^ In our study, we attempted to assess CHK2 and ATM activation, but technical challenges related to antibody sensitivity precluded reliable detection of CHK2 and ATM phosphorylation by Western blot. We therefore focused on CHK1 phosphorylation as a readout for G2/M checkpoint activation consistent with the reduced G2/M fraction observed in mocetinostat‐treated ID3‐KO cells after 24 h of recovery compared to WT cells. The accumulation of cells in G1 can be partially explained by progression from G2/M into G1, where they subsequently arrest. Nonetheless, ATM–CHK2 signaling contributes critically to DSB‐induced checkpoint control and G1 accumulation in this context, and the lack of robust phospho‐ATM/CHK2 detection in our setting represents a technical limitation that should be addressed in future work using optimized phospho‐specific assays.

The observed cell cycle defects explain the reduced proliferation rate, positioning checkpoint collapse as a key driver of synthetic lethality in ID3‐depleted cells treated with HDACi. This selective vulnerability resembles the synthetic lethality observed between class I HDAC inhibition and BRCA1 deficiency, where HDACi treatment similarly induces oxidative stress and DNA damage that preferentially kills BRCA1‐deficient cancer cells with defective HR repair.^42^ Noteworthy, in tumors harboring BRCA1 mutations, HDACi have been shown to downregulate key DNA repair factors, leading to accumulation of unrepaired DSBs and cancer cell death, while sparing normal cells.^42^ However, unlike the study in BRCA1‐deficient cells, our transcriptomic analysis in ID3‐KO cells treated with HDACi did not reveal downregulation of DNA repair genes, suggesting that the impact of HDAC inhibition on DNA repair gene expression is context‐dependent and varies with the genetic background.

Consistent with prior studies demonstrating ID3's role in regulating cell cycle progression and mitotic fidelity,^43^, ^44^ our data reveal that loss of ID3 combined with class I HDAC inhibitor treatment leads to profound transcriptional suppression of genes involved in mitotic division, chromosome segregation, and cell cycle control. These transcriptional alterations align with observed phenotypes of G1 cell cycle arrest and checkpoint exhaustion, providing a coherent mechanistic framework linking ID3 deficiency to the loss of a transcription regulatory axis responsible for mitotic cell division and cell cycle regulation genes under treatment with class I HDACi. The early alterations in gene expression and impaired DNA repair capacity in ID3‐depleted cells, occurring within 48 h from treatment initiation with class I HDACi, precede and likely drive the eventual synthetic lethality.

Our findings support a model in which ID3‐deficient tumor cells exhibit heightened vulnerability to dual impairment of DSB repair mechanisms and subsequent checkpoint collapse. This vulnerability provides a strong rationale for employing HDAC inhibitor‐based therapeutic strategies in tumors deficient in ID3. Support for this concept is reflected in the observed correlation between ID3 loss and downregulation of HR genes in TCGA datasets,^14^ implying that HDAC1/2 inhibitors could effectively target ID3‐low tumors in a similar manner as PARP inhibitors induce vulnerabilities in BRCA‐mutant cancers. This strategy capitalizes on the dual role of ID3 in chromatin remodeling and transcriptional regulation, exploiting tumors that are already primed for catastrophic repair failure and synthetic lethality upon HDAC inhibition.

Our work positions ID3 as a linchpin coordinating chromatin accessibility and distribution of active histone marks at DSBs. The synthetic lethality of ID3 loss and HDAC1/2 inhibition offers a precision medicine strategy for ID3‐deficient malignancies while highlighting the broader role of chromatin dynamics in mediating repair pathway choice. Future studies should explore in vivo efficacy and combinatorial approaches to maximize therapeutic impact.

## AUTHOR CONTRIBUTIONS

**Giuditta Della Corte:** Conceptualization; investigation; formal analysis; methodology; data curation; visualization; software. **Hossam Eldesouky:** Methodology; validation; investigation; formal analysis. **Julia Puchan:** Validation. **Sercan Öz:** Validation. **Elena Everatt:** Data curation; formal analysis. **Ashish Goyal:** Resources; methodology. **Gianluca Sigismondo:** Formal analysis; resources. **Udo Oppermann:** Resources; methodology. **Christoph Plass:** Writing – review and editing. **Dieter Weichenhan:** Writing – review and editing; formal analysis; methodology. **Ali Bakr:** Writing – original draft; writing – review and editing; project administration; supervision; funding acquisition; conceptualization.

## FUNDING INFORMATION

This work is funded by The German Cancer Research Center (DKFZ) and a Grant from the German Research Foundation (DFG, project number 429192355 to A.B.). Research in the U.O. laboratory was supported through funding from Cancer Research UK, the Leducq Epigenetics of Atherosclerosis Network (LEAN) program grant from the Leducq Foundation, and the Myeloma Single Cell Consortium. Research in the G.S. laboratory and mass spectrometry analyses were funded by the German Research Foundation (DFG, project number 446166464).

## CONFLICT OF INTEREST STATEMENT

The authors declare no conflicts of interest.

## Supporting information


**Table S1.** Sequences of oligonucleotides.

**Table S2.** Results of epigenetic drug screening.

**Table S3.** Differentially expressed genes after recovery from HDACi (KO_TvsUT).

**Table S4.** Differentially expressed genes after recovery from HDACi (WT_TvsUT).

**Table S5.** ATAC‐seq sequencing coverage and quality statistics.

**Table S6.** RNA‐seq sequencing coverage and quality statistics.

**Figure S1.** ID3 regulates chromatin accessibility and histone modifications at DSBs.
**Figure S2.** Epigenetic vulnerability screen identifies Class I HDAC dependency.
**Figure S3.** Class I HDAC inhibition exacerbates DNA damage in ID3‐deficient cells.

## Data Availability

High‐throughput sequencing data generated in this study have been deposited to Gene Expression Omnibus (GEO) under the accession numbers GSE301513 and GSE301514. The mass spectrometry proteomics data are available via ProteomeXchange Consortium via the PRIDE^1^ partner repository with the dataset identifier PXD069605. Further information is available from the corresponding author upon request.
