# Supplementary material for: ID3 deficiency alters chromatin accessibility at DSB sites and enhances vulnerability to HDAC inhibition
Source: Int J Cancer. 2026 Feb 24;158(12):3173–86. doi: 10.1002/ijc.70400 (PMC13106931; doi:10.1002/ijc.70400)
Supplement: Supplementary file 7 — Figure S1. ID3 regulates chromatin accessibility and histone modifications at DSBs. Figure S2. Epigenetic vulnerability screen identifies Class I HDAC dependency. Figure S3. Class I HDAC inhibition exacerbates DNA damage in ID3‐deficient cells. [file IJC-158-3173-s007.pdf]

Supplementary Material for

ID3 Deficiency Alters Chromatin Accessibility at DSB Sites and Enhances Vulnerability to HDAC Inhibition

Giuditta Della Corte, Hossam Eldesouky, Julia Puchan, Sercan Öz, Elena Everatt, Ashish Goyal, Gianluca Sigismondo, Udo Oppermann, Christoph Plass, Dieter Weichenhan and Ali Bakr

Table of contents:

Figure S1: ID3 regulates chromatin accessibility and Histone Modifications at DSBs.

Figure S2: Epigenetic Vulnerability Screen Identifies Class I HDAC Dependency.

Figure S3: Class I HDAC Inhibition Exacerbates DNA Damage in ID3-Deficient Cells.

Table S1: Oligonucleotides

Table S2: Results of epigenetic Drug Screening

Table S3: Differentially expressed genes after recovery from HDACi (KO_TvsUT)

Table S4: Differentially expressed genes after recovery from HDACi (WT_TvsUT)

Table S5: ATAC-seq Sequencing Coverage and Quality Statistics

Table S6: RNA-seq Sequencing Coverage and Quality Statistics

All supplementary tables are available in separate excel files.

Figure S1: ID3 regulates chromatin accessibility and Histone Modifications at DSBs. (A & B) Western blot expression analysis of ID3 in WT and ID3-KO cells. (C & D) Cell viability assay showing the proliferation rate and survival analysis, respectively, of WT, ID3-KO cells and ID3-KO cells transduced with ID3 expressing vector, n=3 data are presented as mean ±SD, Student's t test (right panel). (E-G) Western blot expression analysis of HDAC1, HDAC2, and HDAC3 in WT and ID3-KO cells treated with the indicated siRNAs.

Figure S2: Epigenetic Vulnerability Screen Identifies Class I HDAC Dependency. (A & B) Gene set enrichment analysis of the significantly upregulated genes in KO and WT, respectively, after recovery from treatment with class I HDACi. n=3 independent experiments. (C & D) Gene set enrichment analysis of the significantly downregulated genes in KO and WT, respectively, before recovery from treatment with class I HDACi. n=3 independent experiments. (E & F) Gene set enrichment analysis of the significantly upregulated genes in KO and WT, respectively, before recovery from treatment with class I HDACi. n=3 independent experiments.

Figure S3: Class I HDAC Inhibition Exacerbates DNA Damage in ID3-Deficient Cells. (A & B) Volcano plots showing up- and downregulated protein expression in WT and ID3-KO cells, respectively, treated with class I HDACi. (C & D) GO pathway analysis showing the top 10 enriched GO pathways of downregulated proteins in WT and ID3-KO cells, respectively, treated with class I HDACi. (E & F) GO pathway analysis showing the top 10 enriched GO pathways of upregulated proteins in WT and ID3-KO cells, respectively, treated with class I HDACi.

Figure S1

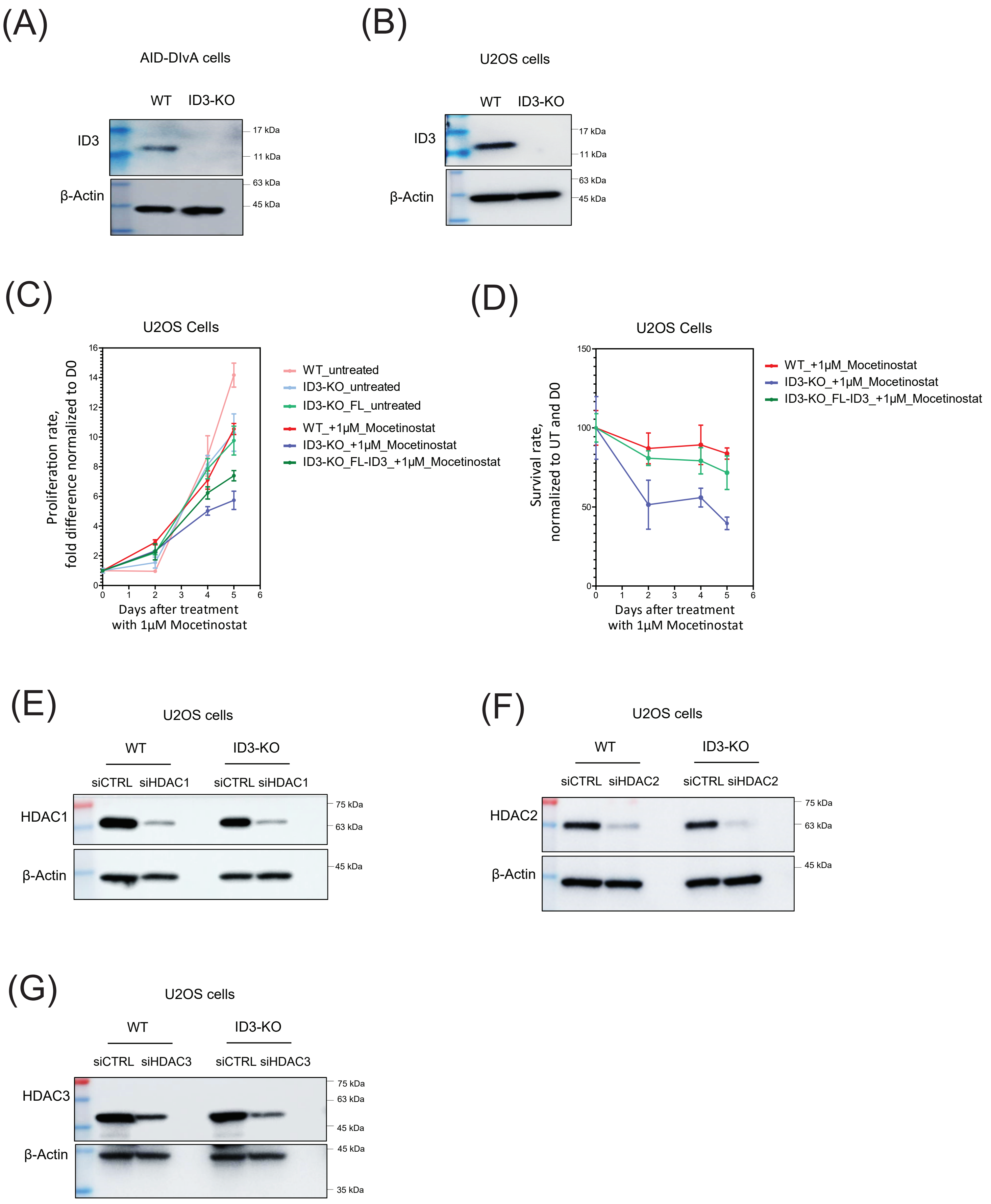

(A)
AID-DIvA cells
WT
ID3-KO
ID3
β-Actin
17 kDa
11 kDa
63 kDa
45 kDa
(B)
U2OS cells
WT
ID3-KO
ID3
β-Actin
17 kDa
11 kDa
63 kDa
45 kDa
(C)
U2OS Cells
Proliferation rate,
fold difference normalized to D0
Days after treatment
with 1µM Mocetinostat
WT_untreated
ID3-KO_untreated
ID3-KO_FL_untreated
WT_+1µM_Mocetinostat
ID3-KO_+1µM_Mocetinostat
ID3-KO_FL-ID3_+1µM_Mocetinostat
(D)
U2OS Cells
Survival rate,
normalized to UT and D0
Days after treatment
with 1µM Mocetinostat
WT_+1µM_Mocetinostat
ID3-KO_+1µM_Mocetinostat
ID3-KO_FL-ID3_+1µM_Mocetinostat
(E)
U2OS cells
WT
ID3-KO
siCTRL siHDAC1
siCTRL siHDAC1
HDAC1
β-Actin
75 kDa
63 kDa
45 kDa
(F)
U2OS cells
WT
ID3-KO
siCTRL siHDAC2
siCTRL siHDAC2
HDAC2
β-Actin
75 kDa
63 kDa
45 kDa
(G)
U2OS cells
WT
ID3-KO
siCTRL siHDAC3
siCTRL siHDAC3
HDAC3
β-Actin
75 kDa
63 kDa
45 kDa
45 kDa
35 kDa

Figure S2

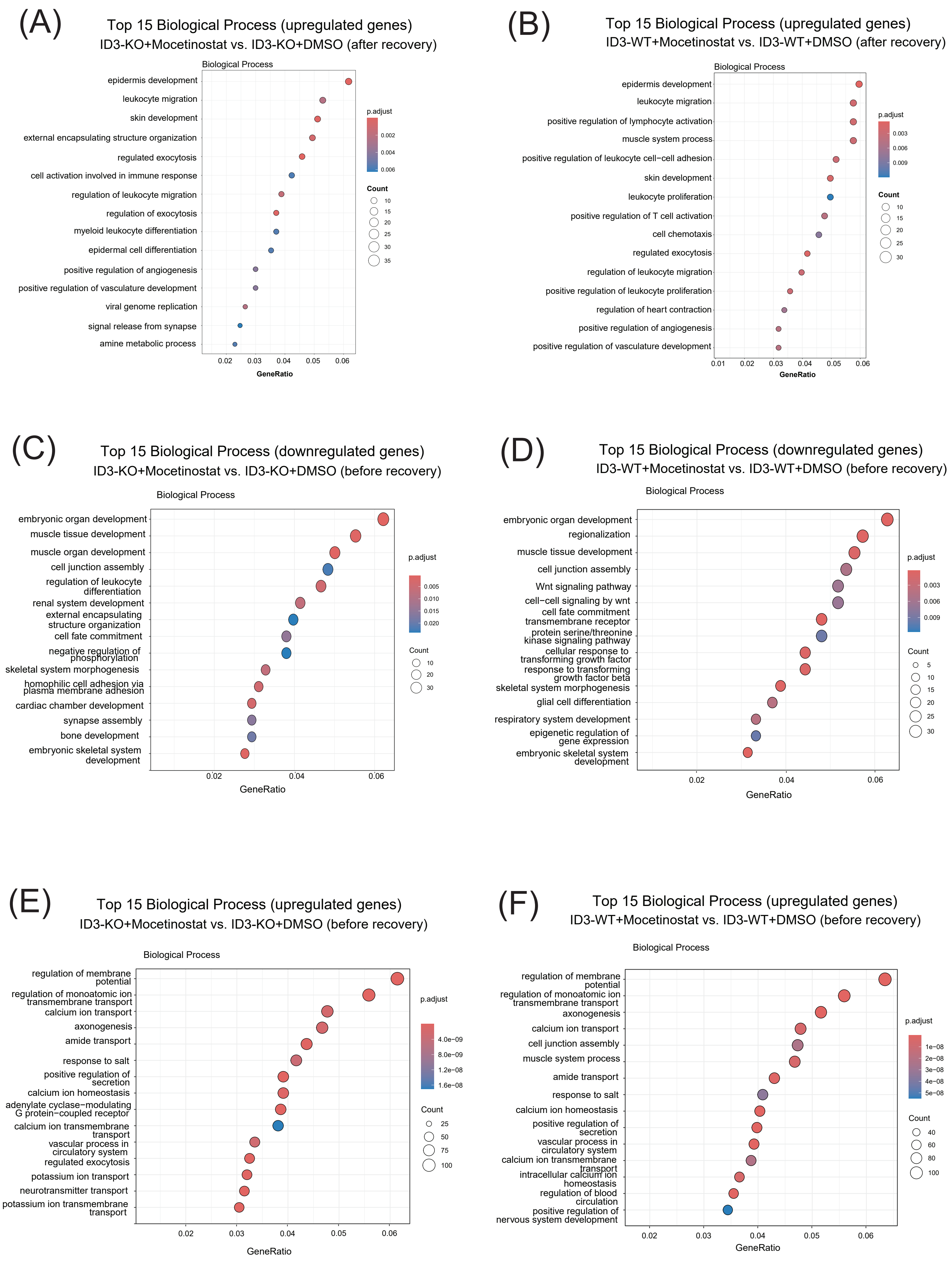
(A)
Top 15 Biological Process (upregulated genes)
ID3-KO+Mocetinostat vs. ID3-KO+DMSO (after recovery)
Biological Process
epidermis development
leukocyte migration
skin development
external encapsulating structure organization
regulated exocytosis
cell activation involved in immune response
regulation of leukocyte migration
regulation of exocytosis
myeloid leukocyte differentiation
epidermal cell differentiation
positive regulation of angiogenesis
positive regulation of vasculature development
viral genome replication
signal release from synapse
amine metabolic process
0.02 0.03 0.04 0.05 0.06
GeneRatio
p.adjust
0.002
0.004
0.006
Count
10
15
20
25
30
35
(B)
Top 15 Biological Process (upregulated genes)
ID3-WT+Mocetinostat vs. ID3-WT+DMSO (after recovery)
Biological Process
epidermis development
leukocyte migration
positive regulation of lymphocyte activation
muscle system process
positive regulation of leukocyte cell−cell adhesion
skin development
leukocyte proliferation
positive regulation of T cell activation
cell chemotaxis
regulated exocytosis
regulation of leukocyte migration
positive regulation of leukocyte proliferation
regulation of heart contraction
positive regulation of angiogenesis
positive regulation of vasculature development
0.01 0.02 0.03 0.04 0.05 0.06
GeneRatio
p.adjust
0.003
0.006
0.009
Count
10
15
20
25
30
(C)
Top 15 Biological Process (downregulated genes)
ID3-KO+Mocetinostat vs. ID3-KO+DMSO (before recovery)
Biological Process
embryonic organ development
muscle tissue development
muscle organ development
cell junction assembly
regulation of leukocyte differentiation
renal system development
external encapsulating structure organization
cell fate commitment
negative regulation of phosphorylation
skeletal system morphogenesis
homophilic cell adhesion via plasma membrane adhesion
cardiac chamber development
synapse assembly
bone development
embryonic skeletal system development
0.02 0.04 0.06
GeneRatio
p.adjust
0.005
0.010
0.015
0.020
Count
10
20
30
(D)
Top 15 Biological Process (downregulated genes)
ID3-WT+Mocetinostat vs. ID3-WT+DMSO (before recovery)
Biological Process
embryonic organ development
regionalization
muscle tissue development
cell junction assembly
Wnt signaling pathway
cell−cell signaling by wnt
cell fate commitment
transmembrane receptor protein serine/threonine kinase signaling pathway
cellular response to transforming growth factor
response to transforming growth factor beta
skeletal system morphogenesis
glial cell differentiation
respiratory system development
epigenetic regulation of gene expression
embryonic skeletal system development
0.02 0.04 0.06
GeneRatio
p.adjust
0.003
0.006
0.009
Count
5
10
15
20
25
30
(E)
Top 15 Biological Process (upregulated genes)
ID3-KO+Mocetinostat vs. ID3-KO+DMSO (before recovery)
Biological Process
regulation of membrane potential
regulation of monoatomic ion transmembrane transport
calcium ion transport
axonogenesis
amide transport
response to salt
positive regulation of secretion
calcium ion homeostasis
adenylate cyclase−modulating G protein−coupled receptor
calcium ion transmembrane transport
vascular process in circulatory system
regulated exocytosis
potassium ion transport
neurotransmitter transport
potassium ion transmembrane transport
0.02 0.03 0.04 0.05 0.06
GeneRatio
p.adjust
4.0e−09
8.0e−09
1.2e−08
1.6e−08
Count
25
50
75
100
(F)
Top 15 Biological Process (upregulated genes)
ID3-WT+Mocetinostat vs. ID3-WT+DMSO (before recovery)
Biological Process
regulation of membrane potential
regulation of monoatomic ion transmembrane transport
axonogenesis
calcium ion transport
cell junction assembly
muscle system process
amide transport
response to salt
calcium ion homeostasis
positive regulation of secretion
vascular process in circulatory system
calcium ion transmembrane transport
intracellular calcium ion homeostasis
regulation of blood circulation
positive regulation of nervous system development
0.02 0.03 0.04 0.05 0.06
GeneRatio
p.adjust
1e−08
2e−08
3e−08
4e−08
5e−08
Count
40
60
80
100

Figure S3

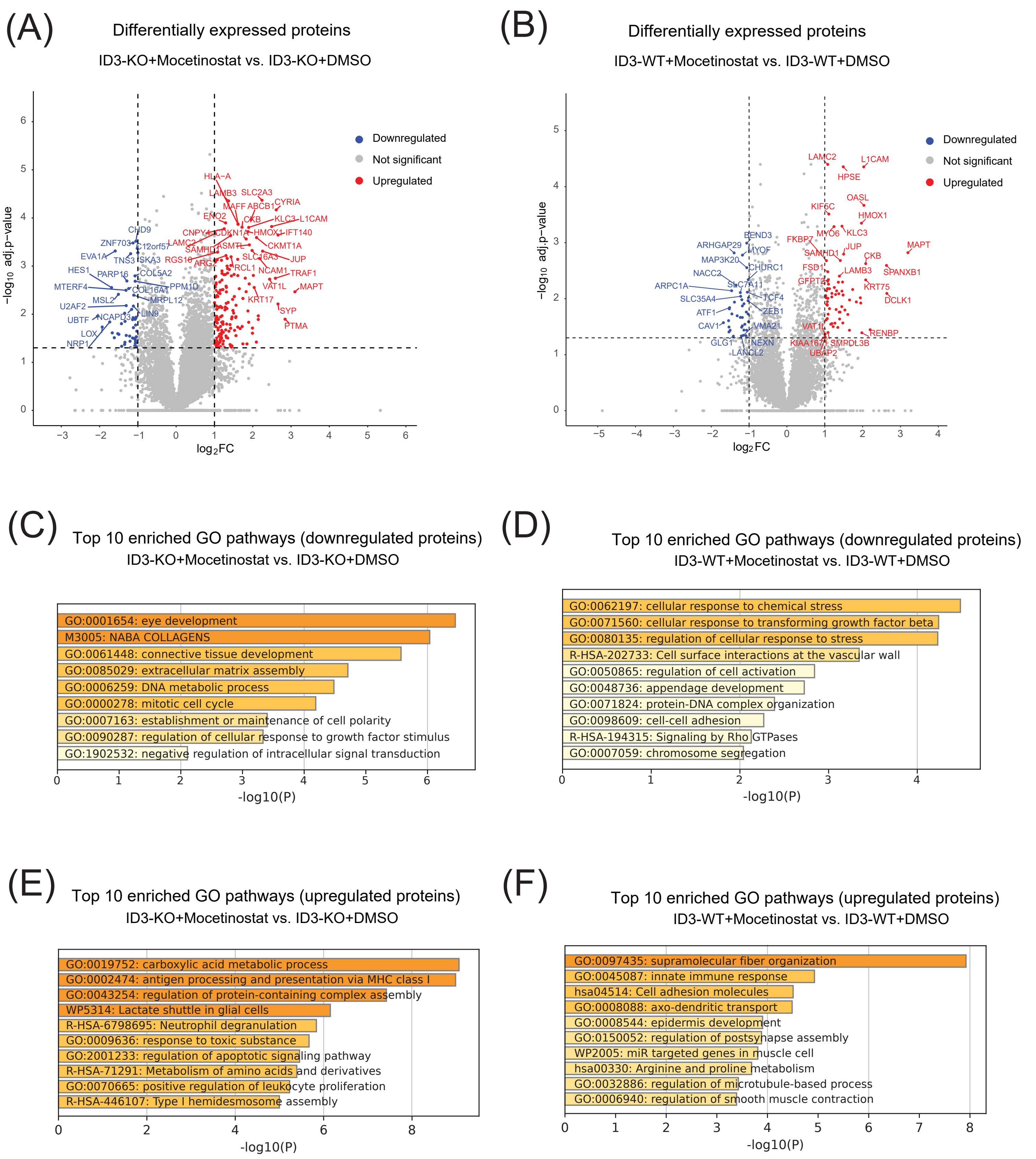
(A)
Differentially expressed proteins
ID3-KO+Mocetinostat vs. ID3-KO+DMSO
−log10 adj.p−value
log2FC
Downregulated
Not significant
Upregulated
(B)
Differentially expressed proteins
ID3-WT+Mocetinostat vs. ID3-WT+DMSO
−log10 adj.p−value
log2FC
Downregulated
Not significant
Upregulated
(C)
Top 10 enriched GO pathways (downregulated proteins)
ID3-KO+Mocetinostat vs. ID3-KO+DMSO
GO:0001654: eye development
M3005: NABA COLLAGENS
GO:0061448: connective tissue development
GO:0085029: extracellular matrix assembly
GO:0006259: DNA metabolic process
GO:0000278: mitotic cell cycle
GO:0007163: establishment or maintenance of cell polarity
GO:0090287: regulation of cellular response to growth factor stimulus
GO:1902532: negative regulation of intracellular signal transduction
-log10(P)
(D)
Top 10 enriched GO pathways (downregulated proteins)
ID3-WT+Mocetinostat vs. ID3-WT+DMSO
GO:0062197: cellular response to chemical stress
GO:0071560: cellular response to transforming growth factor beta
GO:0080135: regulation of cellular response to stress
R-HSA-202733: Cell surface interactions at the vascular wall
GO:0050865: regulation of cell activation
GO:0048736: appendage development
GO:0071824: protein-DNA complex organization
GO:0098609: cell-cell adhesion
R-HSA-194315: Signaling by Rho GTPases
GO:0007059: chromosome segregation
-log10(P)
(E)
Top 10 enriched GO pathways (upregulated proteins)
ID3-KO+Mocetinostat vs. ID3-KO+DMSO
GO:0019752: carboxylic acid metabolic process
GO:0002474: antigen processing and presentation via MHC class I
GO:0043254: regulation of protein-containing complex assembly
WP5314: Lactate shuttle in glial cells
R-HSA-6798695: Neutrophil degranulation
GO:0009636: response to toxic substance
GO:2001233: regulation of apoptotic signaling pathway
R-HSA-71291: Metabolism of amino acids and derivatives
GO:0070665: positive regulation of leukocyte proliferation
R-HSA-446107: Type I hemidesmosome assembly
-log10(P)
(F)
Top 10 enriched GO pathways (upregulated proteins)
ID3-WT+Mocetinostat vs. ID3-WT+DMSO
GO:0097435: supramolecular fiber organization
GO:0045087: innate immune response
hsa04514: Cell adhesion molecules
GO:0008088: axo-dendritic transport
GO:0008544: epidermis development
GO:0150052: regulation of postsynapse assembly
WP2005: miR targeted genes in muscle cell
hsa00330: Arginine and proline metabolism
GO:0032886: regulation of microtubule-based process
GO:0006940: regulation of smooth muscle contraction
-log10(P)
